# Combination of Digital and Conventional Intervention for Sexually Transmitted Infections Prevention among Female Sex Workers

**DOI:** 10.4314/ejhs.v33i5.5

**Published:** 2023-09

**Authors:** Cindy Meilinda Sari, Tri Nurkristina, Bagoes Widjanarko, Ani Margawati

**Affiliations:** 1 Doctoral Program of Public Health, Diponegoro University, Semarang, Indonesia; 2 Faculty of Medicine, Diponegoro University, Semarang, Indonesia; 3 Faculty of Public Health, Diponegoro University, Semarang, Indonesia; 4 Faculty of Medicine, Diponegoro University, Semarang, Indonesia

**Keywords:** Short comedy youtube, Health promotion, STIs prevention, Female sex workers

## Abstract

**Background:**

Female sex workers (FSWs) are at a high risk of contracting sexually transmitted infection (STI). Mobile health (m-health) is one intervention that is easily accessible to everyone online and offline, allowing two-way communication through the program. This study aimed to determine the effectiveness of m-health on STI prevention behavior among FSW.

**Methods:**

The study was conducted using quasi-experimental design. It included the treatment group and the control group, each of which included fifty-four (54) FSWs selected by purposive sampling. The m-health intervention was conducted in the form of a short comedy movie on YouTube, combined with offline assertive communication training to improve skills in negotiating the use of condoms to the intervention group. Meanwhile, the control group received regular counseling from the local community health center. The Mann-Whitney test was used to compare the knowledge, attitudes, motivation, and behavior among the two study groups.

**Results:**

Intervention for six months in the treatment group increased FSWs knowledge score by 4.0 (p=0.00), attitude by 3.9 (p=0.00), and motivation by 12 (p=0.00). The median knowledge, attitude, and motivation scores were 17.38 and 46, respectively. The model is effective onSTI-prevention behavior through motivational mediator variables with a p-value of 0.00. The condom consistency has the highest outer loading value in the STI-prevention behavior construct of 0.71 ≥ 0.4.

**Conclusions:**

A combination of digital and conventional health promotion can improve STI prevention behavior by raising knowledge, attitudes, motivation, and condom consistency behavior.

## Introduction

Sexually transmitted infections (STIs) remain a significant concern due to rising morbidity and mortality rates, which impact people's sexual health and quality of life, particularly in developing nations (1). Contagious infections are caused by bacteria, viruses, or parasites that are spread through sexual contact, including vaginal, anal, and oral sex. The most common sexually transmitted infections are syphilis, gonorrhea, chlamydia, and trichomoniasis (2). Long-term consequences of untreated STIs include infertility, cervical cancer,ectopic pregnancy, salpingitis, and pelvic pain. Southeast Asia has a high STI prevalence, with 156 million people suffering from trichomoniasis, 82 million from gonorrhea, 128 million from chlamydia, and 7 million from syphilis. The spread of these diseases is concentrated in key populations, especially among FSWs (3).

Bali is one of the provinces in Indonesia with the highest number of STI cases in FSW; in 2016, it reached 60% of the total STI cases (4). The most common STIs were chlamydia (32.3%), gonorrhea (21.2%), and syphilis (6.5%) (5). Buleleng is a district of Bali province that ranks the second-highest in number of STI cases (6). In 2021–2022, STIs among FSW cases were quite significant at 72%. Cervicitis had the highest occurrence (147 cases), followed by syphilis (139 cases), and gonorrhea (81 cases), according to a baseline community-based screening (7).

Female sex workers have a high risk of exposure to STIs and contribute significantly to the spread of STIs (8). The risk factor for STI transmission in FSWs is risky sexual behavior, i.e., the inconsistent use of condoms, which is an obstacle to preventing STIs (9). Lack of knowledge about STIs results in ineffective preventive behavior, such as the common practice among FSWs to perform vaginal douching after sexual activity (10). This behavior has the effect of 59.4% irritation which can increase STIs and HIV (11). Currently, efforts to prevent STIs in Indonesia, apart from using condoms, also focus on testing and health education designed to reduce risky behavior (12). Behavior change interventions in critical populations are carried out through an outreach system by NGOs (13). The program is funded internationally, and sustainability still needs to be considered (14). Prior interventions of health promotion, efforts to prevent risky behavior, case finding (screening, testing, tracing), and case management have not been widely successful (13). This is evidenced by the low rate of consistent condom use, the high rate of STIs, and the presence of new infections among FSWs (15).

The use of technology to improve health prevention behavior has been expanding globally as people increasingly rely on the internet as a source of health information including FSWs who often access youtube (16-18). YouTube is a website that allows individuals and organizations to upload and share video content (19). Social media provides a way to disseminate health information that has proven effective in supporting the promotion of health and allowing the formation of virtual communities—in particular among populations often marginalized by society, such as FSWs—to freely and anonymously share experiences on sensitive topics, such as sexual activity (20). This study aims to develop an effective health promotion model combining digital and conventional media to increase the independence of FSWs in increasing STI-prevention behavior.

## Materials and Methods

**Design and research subject**: A combination of digital and conventional health promotion models was given to FSWs as part of an experimental study. Their effectiveness was tested using a quasi-experimental approach. The subjects were selected purposively from data on Buleleng Community Health Center, Bali, Indonesia. The research was conducted from January to August 2022. The subjects were selected purposively: out of a total of 108 sex workers, 54 respondents for the intervention group and 54 respondents for the control group were used. The inclusion criteria for FSWs were having a smartphone with the YouTube app, being willing to not move for six months, and agreeing to participate in the study. The exclusion criterion was being unwilling to be tested for STIs or HIV.

**Intervention**: A combination of digital and conventional intervention was used: YouTube content consisting of 12 comedic videos and offline condom negotiation training. YouTube content is developed by the research team and validated by material and media experts such as public health lecturers, health promotion practitioners, content creators, and communication science lecturers. YouTube intervention was carried out for 12 weeks, with one video shown per week. In addition, the group received 12 weeks of training, with one training session consisting of 120 minutes of offline condom negotiation instruction per week. The control group received regular outreach without media from the local community health center. All five STI program holders with a non-governmental organization Bali Foundation have set schedules for visiting the site of the control group quarterly as part of mobile VCT activities. STI providers distribute free condoms and convey information about the importance of condom use when having sexual intercourse with regular partners and customers.

**Data collection and follow-up**: Data collection was conducted through interviews and observation. Instruments in the form of questionnaires were tested for validity and reliability. Data collection was divided into three stages applicable to the intervention and control groups, namely: before intervention (pre-test I), after three months of intervention (post-test I), and three months after the post-test I (post-test II). Measurements were used to assess knowledge, attitude, motivation, and behavior.

**Sample size**: Sample size is 54 pairs for each group based on the minimal sample size determined by WHO for health studies.

**Variables**: The dependent variables include knowledge, attitudes, motivation, and practices of 1) condom consistency, 2) personal hygiene, 3) STI screening, and qa4) assertive communication.

**Data processing and analysis**: Data were checked for completeness and analyzed using the Statistical Package for Social Science (SPSS) version 21.0. The validity test was run with SPSS 21 utilizing the Pearson product moment test and the Cronbach Alpha approach was used to assess reliability. Analysis used a comparative test before and after using the Mann-Whitney, and the effect of intervention by using partial least squares (SEM-PLS).

**Ethics**: All subjects in this study gave informed consent. Ethical clearance was obtained from the Ethical Commission of Public Health Faculty, Diponegoro University, with the number 26/EA/KEPK-FKM 2022.

## Results

**Individual characteristics of the respondents**: Table 1 shows the highest proportion in the control and intervention groups, namely at 20–35 years old. In addition, Table 1 demonstrates that the results of the homogeneity test showed that the significance value is > 0.05, which means that the control and intervention group respondents had the same characteristics in the variables of age, education, length of work, history of STIs, and marital status.

**Table 1:** Subject characteristics

Variable	Category	Intervention		Control		*p value*
			
		n	%	n	%	
Age	< 20 years	9	16.7	7	13	
	20–35 years	37	68.5	37	68.5	0.16
	>35 years	8	14.8	10	18.5	
Education	Elementary school	10	18.5	6	11.1	
	Junior high	32	59.3	29	53.7	0.19
	Senior high	12	22.2	19	35.2	
Length of Work	< 6months	5	9.3	15	27.8	
	6months–2years	31	57.4	22	40.7	0.08
	>2 years	18	33.3	17	31.5	
History of STIs	Yes	34	63	30	55.6	
	No	20	37	24	44.4	0.06
Marital Status	Single	11	20.4	10	18.5	
	Married	15	27.7	19	35.2	0.90
	Divorced	28	51.9	25	46.3	

* significant at *p* <0.05

**The effect of intervention on knowledge, attitude, motivation, and behavior**: Table 2 shows the effect of the intervention on STIs prevention (p=0.00; δ=4.0) after a six-month intervention as well as the attitude toward the prevention of STIs (p=0.00; δ=3.9) and motivation (p=0.00; δ= 12.4). There was a significant relationship between knowledge, attitude, motivation, and behavior before and after intervention with p < 0.05. An increase in knowledge, attitude, motivation, and behavior after six months in both the intervention and control groups was seen in the median value.

**Table 2:** The Effect of Intervention on Knowledge, Attitude, Motivation, and Behavior

Variable	Intervention group		Control group			

	Median (min-max)	Mean (SD)	Median (min-max)	Mean (SD)	Score Difference	*p*
Knowledge						
Pre	14.0 (4-20)	13.2 (3.75)	14.0 (5-20)	13.5 (3.10)	-0.3	0.88
Post I	14.0 (8-20)	13.9 (2.68)	14.0 (5-20)	13.5 (3.10)	0.4	0.77
Post II	17.0 (13-21)	17.4 (2.40)	14.0 (8-20)	13.5 (3.10)	4.0	0.00*
Attitude						
Pre	34.0 (27-41)	34.0 (3.74)	34.0 (27-42)	35.0 (3.40)	-1	0.18
Post I	35.0 (26-42)	35.0 (3.58)	34.0 (26-42)	35.1 (3.59)	0.9	0.68
Post II	38.0 (31-43)	38.0 (3.22)	36.0 (31-43)	35.8 (2.94)	3.9	0.00*
Motivation						
Pre	32.0 (24-52)	33.5 (4.53)	32.0 (24-52)	33.3 (2.34)	0.2	0.94
Post I	32.0 (28-52)	33.5 (4.53)	32.0 (26-46)	32.4 (2.08)	1.0	0.46
Post II	46.0 (41-50)	45.9 (2.54)	33.0 (29-40)	33.8 (2.18)	12.4	0.00*

* significant at *p* <0.05, pre=before intervention, post I= after 3 months intervention, post II=after 6 months intervention

**Figure 2:**
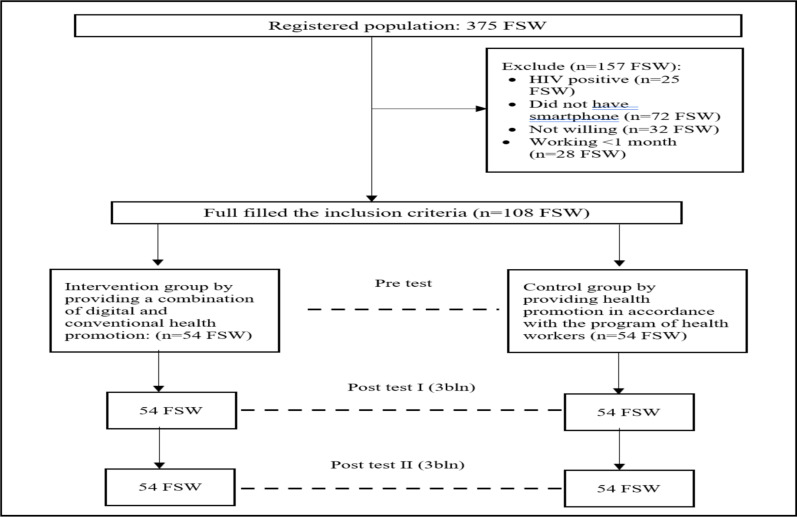
Consort diagram

**The effect of the intervention on FSW behavior SEM-PLS**: Figure 2 and Table 3 show that all indicators have an outer loading value of ≥ 0.4, so they pass the validity test. Furthermore, all construct variables have passed the reliability test because they have a composite value of ≥ 0.6. For example, the condom consistency behavior indicator has the highest outer loading value in the STI-prevention behavior construct of 0.71 ≥ 0.4.

**Table 3:** Outer Loading, AVE and Composite Values

Variable	Indicator	Code	Outer	Composite	R-Square
Construct			Loading		Adjusted
Motivation	External motivation	M_EKS	0.92	0.91*	0.87
	Internal motivation	M_IN	0.91		
Model	Digital and	Model	1.00	1.00*	-
	conventional				
	combination model				
STI prevention behavior	Condom consistency	PLKU_KKON	0.69	0.74*	0.44
	Personal hygiene	PLKU_P_HGINE	0.70		
	Sexually transmitted	PLKU_P_SKRIN_IMS	0.62		
	infection screening				
	Assertive	PLKU_P_KOMASER	0.57		
	Communication				
Knowledge	Condom consistency	PENGT_PKP_TTL	0.68	0.69*	0,39
	Personal hygiene	PENGT_PORW_TTL	0.41		
	Sexually transmitted	PENGT_TG_IMS_TTL	0.41		
	infection screening				
	Assertive	PENGT_KOM_ASER	0.27		
	Communication				
Attitude	Attitude	SKAP	1.00	1.00*	0.26

* significant at composite > 0.6

The next stage is testing the structural model. The adjusted R-Square value for STI-prevention behavior is 0.44. STI-prevention behavior variability (X11) is explained by motivation (X4) and knowledge (X2) of 43.1% The R-Square value of 0,43 indicates that the model is moderate. The motivation has an R-Square value of 0.87, which demonstrates that the variability of motivation is explained by the combined digital and conventional health promotion model of 87%. The R-Square value of 0.87 indicates that the model is good.

Table 4 shows that the digital and conventional combination of health promotion model has a significant effect on knowledge t-statistic value of 13.69 ≥ 1.96 and p value of 0.00 < 0.05. The digital and conventional combination of health promotion model significantly affects attitudes because it has a t-statistic value of 6.17 ≥ 1.96 and p-value of 0.00 < 0.05. Furthermore, the digital and conventional combination health promotion model has a significant effect on STI-prevention behavior through motivational mediator variables because it has a t-statistic value of 6.11 ≥ 1.96 and p-value of 0.00 < 0.05.

**Table 4:** Path Coefficients

Relationship between variables	Original Sample	T Statistics	*P* Values
Digital and conventional combination health promotion models -> knowledge	0.63	13.69	0.00*
Digital and conventional combination health promotion models -> attitude	0.53	6.17	0.00*
Digital and conventional combination health promotion models -> motivation	0.93	42.66	0.00*
Knowledge -> behavior	-0.07	0.58	0.56
Knowledge -> attitude	-0.03	0.33	0.74
Attitude -> motivation	0.01	0.40	0.69
Motivation -> STI-prevention behavior	0.60	6.18	0.00*
Digital and conventional combination health promotion models -> motivation	0.56	6.11	0.00*
-> STI-prevention behavior			

* significant at *p* <0.05

## Discussion

A combination of digital and conventional intervention increased the knowledge of FSWs after three and six months. The intervention was carried out every week using a short comedy movie on a YouTube channel accompanied by conventional training to improve condom negotiation skills, raise awareness, improve understanding, and encourage positive behavior changes among FSWs. Prevention efforts were conducted using media, which is easily accessible, engaging, and suitable for FSW (4). Several studies state that providing health information using audiovisual media effectively increases knowledge (21). Selecting the right method, using targeted media, and the topics presented all contribute to the successful implementation of health promotion (22). Advances in technology and information, including the internet, have led to increases in life expectancy (23). Internet access is fundamental to the effective digitization of health programs (24). STI-prevention programs for female sex workers that utilize digital technology and are implemented through social media have been effective in increasing STI-prevention behavior in several countries (25).

There are differences in the attitudes of female sex workers about preventing STIs before and after being given a combination of digital and conventional health promotion interventions. This difference was demonstrated by the mean value of the pre-test results of the intervention group during the pre-test and post-test after being given a combination of digital and conventional health promotion interventions. The results showed that the health promotion model given to FSWs is useful for increasing positive attitudes in preventing STIs. Another research also shows that there is a change in individual attitudes from negative to positive (26).

Attitude is a set of symptoms in response to a stimulus. The response will occur if the individual reacts to a stimulus, where the reaction is expressed as an attitude that manifests as the result of an internal evaluation process. This study proves that FSWs responds well to the chosen stimulus. Each individual has a different attitude toward different stimuli and may react based on their judgments of whether a particular stimulus is good/bad, pleasant/unpleasant, or important/unimportant. Individuals have positive traits when they feel happy and can place them at the level of existing attitudes. Health promotion is a stimulus that influences individual thoughts and attitudes (27).

The motivation of female sex workers in the intervention group to prevent STIs increased by 12.5 from 30.52 to 43.02 after they were administered a combination of digital and conventional health promotion. The group displayed differences in motivation before and after being given a combination of digital and conventional health promotion interventions. Motivation is an internal condition that prompts a person to act, achieve goals, and show interest in certain activities. Motivation consists of intrinsic motivation, which is influenced by needs, expectations, and interests; and extrinsic motivation, which is influenced by media factors, sexual partner support, and health information.

Someone who is self-motivated will find it easier to achieve success compared to someone for whom motivation depends on external driving factors. Individuals who are intrinsically motivated have initiative and are actively trying to learn or improve, so understanding and insight about STI prevention come easier to these people than to those who are more passive and less internally motivated. The internal factor that influences motivation is knowledge. Individuals with a good knowledge base tend to have positive values, attitudes, and motivation towards healthy living principles and have skills in carrying out matters related to health care (28).

An educational approach to health promotion is one of the best ways to provide information and motivation to encourage appropriate attitudes and actions. Health promotion is an external factor that can influence the emergence of a person's internal motivation. Experience and education can affect whether the information is accepted and transformed into motivation. Female sex workers who do not experience an increase in motivation after receiving the combined health promotion may have a different level of information acceptance.

Female sex workers in the intervention group displayed differences in motivation to prevent STIs before and after being given a combination of digital and conventional health promotion interventions. The results of the post-test on the control group showed that FSWs in the high-motivation category did not experience a change in the level of motivation to prevent STIs. A person's behavior can be influenced by reinforcing factors, namely motivation from other people, such as partners. Social support has a positive impact on individuals from other people in their social environment in verbal and nonverbal forms, such as attention, affection, advice, and judgment.

Support from sexual partners in this study, namely providing information on STI prevention (condom use, STI testing, intimate organ care), providing condoms, and always advocating using condoms when having sexual intercourse, causes female sex workers to have high motivation to be consistent in using STI-prevention methods. Open disclosure of information about the importance of using condoms consistently as an effort to prevent STI transmission can provide a mutually beneficial outcome for sex partners in preventing STI transmission (29).

The STI-prevention behavior of female sex workers in the intervention group after being given combined digital and conventional health promotion showed a mean value (14.24) greater than the mean value before being given health promotion (8.54), showing that there was a significant change in STI-prevention behavior after being given combined digital and conventional health promotion. The digital and conventional combination of health promotion model has a significant effect on STI-prevention behavior through motivational mediator variables because it has a t-statistic value of 6.444 ≥1.96 or a p-value of 0.000 <0.05.

Information, motivation, and behavior skills are the main factors that can influence individual STI-prevention behavior (30). The risk of transmission can be prevented more easily through increased information, motivation, and skills. The information referred to relates to knowledge about STIs and preventive measures. Skill is the ability to take preventive actions such as practicing safe sex (31).

Behavioral skills are prerequisites that determine whether good information and motivation can influence behavior change in effective STI prevention (32). In this case, information about STIs can influence a person's sexual behavior (33). Interventions carried out in several countries such as Brazil, Nepal, and Tanzania have proven that being well-informed greatly influences STI-prevention behavior among female sex workers (8,22). Having a high level of knowledge about STIs, including symptoms, modes of transmission, screening, and treatment, encourages the practice of safe sex (34). The behavior of using condoms consistently, which was lacking before being given health education, is caused by the low level of condom negotiation, especially with regular partners (35). The condom use rate with regular partners is only 24.1% (36).

Consistent use of condoms is an effective preventive behavior to avoid STIs. Scientific evidence shows that proper and continuous use of condoms can prevent more than 95% of STI cases. A 2016 study in Ethiopia found that only 46.5% of female sex workers used condoms and that respondents who did not use condoms had a risk level for contracting an STI that was four times higher than those who used condoms. This indicates that one out of five female sex workers in the city of Finote Selam is infected with an STI (37).

STI control is measured by reducing the incidence and prevalence, which is achieved by implementing a strategy consisting of the synergy of interventions. In-depth interviews conducted with STI program holders and female sex workers indicated that a training program to improve safe sex behavior in preventing STIs had never been done before. The digital and conventional combination of health promotion models that are applied to female sex workers, apart from providing health information about STI prevention, provide training related to STI prevention, which includes negotiating condoms, how to care for intimate organs, and improving internal support systems in STI screening. The skills training provided as an intervention to promote STI prevention is effective in increasing STI-prevention behavior in female sex workers (38). Green states that individual healthy behavior is influenced by predisposing, enabling, and reinforcing factors. The results of this study illustrate that the skills of female sex workers in preventing STIs are predisposing factors that support shaping individual behavior.

The disinterest or reluctance of female sex workers to participate in health and welfare programs appears to be a major barrier to changing health behavior. The use of media in the implementation of health promotion aims to attract interest and make it easier for the target group to understand the information conveyed. The information media in this intervention is an audiovisual type that is disseminated via social media. Anderson revealed that the use of audiovisual media stimulates interest in listening, which improves concentration (39). This strategy can reduce boredom and enable individuals to better comprehend the message conveyed. Respondents who were not supported by the media had a 21 times greater chance of not using condoms than those who received support from the media (40).

In conclusion, our study showed that FSWs' knowledge, attitude, motivation, and behavior increased significantly in six months after combining digital and conventional interventions to prevent STIs. Therefore, health promotion using YouTube is recommended to improve FSWs' prevention of STIs.

## Figures and Tables

**Figure 1:**
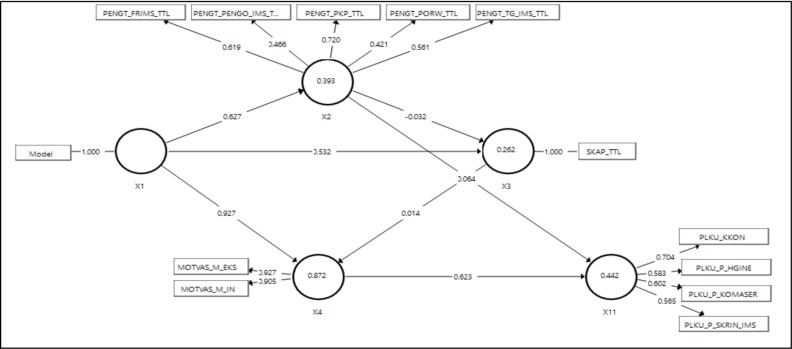
The effect of intervention knowledge, attitude, motivation, and behavior
